# Muscle–tendon unit length changes differ between young and adult sprinters in the first stance phase of sprint running

**DOI:** 10.1098/rsos.180332

**Published:** 2018-06-13

**Authors:** Jeroen Aeles, Ilse Jonkers, Sofie Debaere, Christophe Delecluse, Benedicte Vanwanseele

**Affiliations:** 1 KU Leuven - University of Leuven Department of Kinesiology, Human Movement Biomechanics Research Group, 3000 Leuven, Belgium; 2 KU Leuven - University of Leuven Department of Kinesiology, Physical Activity, Sports and Health Research Group, 3000 Leuven, Belgium

**Keywords:** ratio of forces, joint stiffness, musculoskeletal modelling, performance, well-trained athletes

## Abstract

The aim of this study was to compare young and adult sprinters on several biomechanical parameters that were previously highlighted as performance-related and to determine the behaviour of several muscle–tendon units (MTU) in the first stance phase following a block start in sprint running. The ground reaction force (GRF) and kinematic data were collected from 16 adult and 21 young well-trained sprinters. No difference between the groups was found in some of the previously highlighted performance-related parameters (ankle joint stiffness, the range of dorsiflexion and plantar flexor moment). Interestingly, the young sprinters showed a greater maximal and mean ratio of horizontal to total GRF, which was mainly attributed to a greater horizontal GRF relative to body mass and resulted in a greater change in horizontal centre of mass (COM) velocity during the stance phase in the young compared with the adult sprinters. Results from the MTU length analyses showed that adult sprinters had more MTU shortening and higher maximal MTU shortening velocities in all plantar flexors and the rectus femoris. Although previously highlighted performance-related parameters could not explain the greater 100 m sprinting times in the adult sprinters, differences were found in the behaviour of the MTU of the plantar flexors and rectus femoris during the first stance phase. The pattern of length changes in these MTUs provides ideal conditions for the use of elastic energy storage and release for power enhancement.

## 1. Introduction

Sprint performance depends on an effective start followed by achievement and maintenance of the highest possible running velocity [1]. To do this, sprinters must accelerate their body centre of mass (COM) in a forward direction at maximal effort. As the highest COM acceleration during a sprint occurs during the first stance phase [2], it is not surprising that the ability to generate maximal external power during this phase is linked to overall sprint performance [3,4]. After block clearance, the first stance phase is crucial for transferring the power generated during the block start to the acceleration phase. Therefore, the first stance phase has been highlighted as a critical segment of the acceleration phase and studying this will increase our overall knowledge about sprint performance.

Previous research has highlighted the important contribution of several biomechanical parameters during the first stance phase to sprint performance. These parameters include a high ratio of forces (RF) in percentage of horizontal to resultant ground reaction force (GFR) [5,6], high ankle joint stiffness [7] and a reduction in range of dorsiflexion, which requires high plantar flexor moments [8]. All of these studies have drawn their conclusions based on the testing of well-trained and even elite adult sprinters. In a recent study, Debaere *et al*. [9] have shown that there is a significant difference between young and adult well-trained sprinters in specific technical skills. They highlighted a difference in relative joint power contribution to total power generation between the two groups. The adult sprinters relied more on knee joint power generation, whereas the young sprinters had more relative contributions from their hip joint [9].

The altered relative contributions of joint power in the young athletes compared to the adult athletes shown by Debaere *et al*. [9] may indicate a less optimal inter-joint coordination compared with adult sprinters contributing to less efficient acceleration during the first step of sprint running. As the knee joint power contribution in the adult group was much higher compared with the young athletes, the bilateral gastrocnemius muscles may present a longer stretching time and higher magnitudes of stretching of the muscle–tendon unit (MTU), possibly increasing their potential for energy storage [10]. Furthermore, this higher knee joint contribution to power generation in the adult sprinters may result in more and earlier shortening of the bilateral rectus femoris during knee extension. The young athletes, on the other hand, might experience longer periods of stretching the rectus femoris and stretching it more due to their higher contribution of hip joint power generation. This difference in timing and magnitude of stretching and shortening of the MTUs, mainly the bi-articular MTUs, may have important consequences for their elastic energy behaviour and negatively impact the ability to accelerate the body COM in the young sprinters. Indeed, Bobbert & van Ingen Schenau [11] argued that a proximal to distal power transfer is crucial for maximizing energy used for accelerating the body COM and highlighted the important role of bi-articular MTUs in this transfer of power. The impaired inter-joint coordination observed in the young athletes may, therefore, suggest suboptimal MTU behaviour, especially in bi-articular muscles responsible for energy transfer between joints. This may in turn limit the COM acceleration. To the best of our knowledge, no study investigated the behaviour of individual MTUs that span both ankle, knee and hip joint during the first stance phase in sprint running as these cannot readily be derived from joint kinematics but rely on the use of a musculoskeletal model that accounts for the individual muscle moment arms at the different joints.

It remains unknown whether differences in the other aforementioned performance-determining parameters, more specifically RF, ankle joint stiffness and the range of dorsiflexion, are present between adult and young sprinters. We hypothesized that the young sprinters would have a lower RF, lower ankle joint stiffness and an increased dorsiflexion range of motion (ROM) accompanied by a lower plantar flexor moment, compared with the adult sprinters. Owing to the different joint coordination patterns in terms of power generation between young and adult sprinters, we expected differences in the timing and magnitude of MTU length changes, mainly in the bilateral muscles indicative of impairment of the energy storage and release cycle. Understanding what skills specific to the first stance phase young sprinters lack compared to adult sprinters can greatly help coaches improve performance at early ages.

## 2. Methods

### 2.1. Participants

Two groups of athletes were included in this study. The first group consisted of 16 adult well-trained Belgian sprint athletes (seven male: 24.4 ± 2.4 years; 172.2 ± 5.3 cm; 77.5 ± 7.6 kg; personal best 100 m: 10.67 ± 0.14 s and nine female; 23.4 ± 3.4 years; 163.8 ± 6.1 cm; 59.4 ± 4.9 kg; personal best 100 m: 12.12 ± 0.41 s), whereas the second group consisted of 21 well-trained young athletes (11 male: 16.5 ± 1.6 years; 177.9 ± 7.8 cm; 64.7 ± 7.5 kg; personal best 100 m: 11.47 ± 0.34 s and 10 female: 16.7 ± 1.4 years; 171.5 ± 5.6 cm, 57.5 ± 6.3 kg; personal best 100 m: 12.75 ± 0.36 s). All subjects gave their written informed consent. This study conforms to the recommendation of the Declaration of Helsinki and has been approved by the local ethics committee (UZ Leuven).

### 2.2. Procedures

All athletes were tested three to six weeks prior to major competitions, ensuring that all athletes were in good form and not fatigued from competition. Testing took place in an indoor sports hall on a tartan track surface. Athletes wore regular training clothing and their own running spikes and were allowed to adjust the starting blocks to their own preferences. After an individual warm-up, each athlete performed three maximal effort 10 m sprints with 6 min recovery time between the sprints. Normal competition starting procedures were used.

### 2.3. Data collection: optoelectronic motion capturing

A three-dimensional motion analysis system (Vicon, Oxford Metrics, UK) was used to capture the motion during the first stance phase after block clearance using 12 MX3 cameras (250 Hz, 325 000 pixels). Each subject was equipped with 74 spherical reflective markers, containing eight technical clusters. Eight medial markers were removed after the initial static trial in the T-position to avoid movement interference. A Kistler force plate, embedded in the track (1000 Hz), was used to measure the GRF of the first step.

### 2.4. Data processing

**Figure 1.**
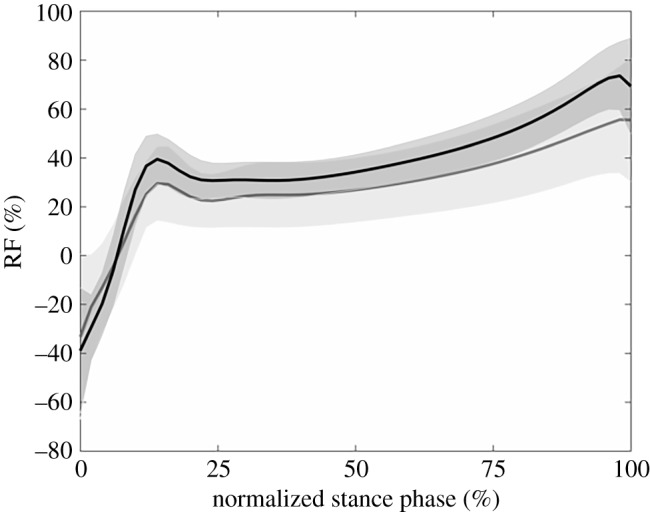
Ratio of forces (RF) in percentage of horizontal to resultant GRF for the young (black line) and adult (grey line) sprinters. Shaded areas represent 1 s.d.

RF was calculated as the percentage of the sagittal plane GRF contributing to the three-dimensional resultant GRF. GRFs were first low-pass filtered using a fourth-order Butterworth filter at 30 Hz [6] and normalized to 50 data points for group average calculations. Maximal and mean RF of the first stance phase were calculated as suggested by Morin *et al*. [6]. Furthermore, because the first stance has a significant breaking phase during which the resultant GRF is oriented in posterior direction, we also calculated the minimal (negative) RF values for each subject. Figure 1 shows that it is only a small portion of the full stance phase during which negative RF values are present. Furthermore, as Morin *et al*. [5,6] have shown that horizontal but not resultant GRF is related to sprint performance, we compared the maximal and mean horizontal component of the GRF during the first stance between both groups.

Following initial marker labelling in Nexus (Vicon, Oxford Metrics, UK) a 14 segment (head, trunk (HT), pelvis and bilateral upper arm, lower arm, hand, femur, tibia and foot), 26 d.f. model with 92 MTU actuators [12] was used (OpenSim 1.9) [13]. The model was first scaled to the individual anthropometry of the athlete, based on marker positions obtained during the static trial and the subject mass. Then, using the three-dimensional marker trajectories of the sprint trial, an inverse kinematics procedure calculated the joint angles along the different d.f.s of the model. Joint angles were then used as input for a three-dimensional inverse dynamics calculation to calculate the internal net moments at each of the lower limb joint. After calculation, joint angles and joint moments were low-pass filtered (12 Hz) using a fourth-order Butterworth filter that was implemented in Matlab (version R2013, Mathworks) to adequately suppress motion artefacts without inducing excessive smoothing of the traces. Joint moments were normalized to body mass. As the ankle and knee joint presented only plantar flexor and knee extensor moments during the stance phase, only their maximum was compared between groups. The hip joint moment changes from a hip extensor to a hip flexor moment throughout the stance phase [9,14], as such both minimal (further referred to as maximal hip flexor) and maximal (further referred to as maximal hip extensor) moments were compared.

Joint stiffness of the ankle during the first stance phase was calculated as the linear regression coefficient of the joint moment–angle curve. Calculation of total joint stiffness over the entire stance phase resulted in only moderate determination coefficients ($R_{\mathrm{Ankle}}^{2}<0.70$). Therefore, as proposed by Butler *et al*. [15], joint stiffness was calculated during two separate phases: during the increase in joint moment and during the decrease in joint moment. Using this method, good determination coefficients were obtained ($R_{\mathrm{Ascending};\;\mathrm{ankle}}^{2}>0.98$; $R_{\mathrm{Descending};\;\mathrm{ankle}}^{2}>0.95$). The phase during which joint moment increases will further be addressed as the ascending limb of the moment–angle curve. The phase during which the joint moment decreases will be referred to as the descending limb.

MTU lengths from seven muscles of the stance leg (soleus, medial gastrocnemius, lateral gastrocnemius, rectus femoris, vastus lateralis, vastus medialis and vastus intermedius) were calculated by conducting a muscle analysis procedure in OpenSim 1.9. Reference MTU lengths were calculated with each scaled model placed in anatomical position (i.e. all angles equal 0°). The lengths of the MTUs were scaled in the scaling step of this workflow [12]. Then, for each participant, these reference MTU lengths were subtracted from the MTU lengths of the first stance phase. For each MTU, the maximal length increase (further addressed as stretching) and decrease (shortening), the absolute time duration of the stretching and shortening and the maximal shortening velocities were calculated. The velocity was calculated as the time derivative of the MTU length changes throughout the stance phase.

Contact time was calculated as the time between initial foot-ground contact (vertical GRF > 20 N) and toe off (vertical GRF < 20 N). The change in COM horizontal velocity during the stance phase was calculated as the horizontal impulse, i.e. the area under the curve of the horizontal GRF normalized to body mass in function of time. Correlation coefficients were calculated to determine the relationship between the change in COM horizontal velocity during the stance phase and all parameters that were compared between the groups, as specified in the statistical analyses section of the methods. For this, both the data from the youth and the adult group were combined as one dataset, in order to increase the power of these calculations.

### 2.5. Statistical analyses

Statistical analyses were performed using Matlab (v. R 2013, Mathworks). Normality of the data was confirmed based on skewness values lower than −1 or greater than 1 and all data were compared using parametric tests. Contact time, change in COM horizontal velocity during the stance phase, minimal, maximal and mean RF, horizontal GRF, ankle joint stiffness, ankle joint dorsiflexion range, minimal, maximal and ROM of ankle, knee and hip joint angles and minimal hip flexion moment and maximal ankle, knee and hip joint moments, absolute MTU stretching length changes, absolute MTU stretching time, absolute MTU shortening length changes, absolute MTU shortening time and maximal MTU shortening velocities were compared between groups using a one-way ANOVA. A one-way ANOVA was also used to compare the ankle joint stiffness on the ascending and descending limb. Correlation coefficients were calculated using the Pearson product-moment test. Only significant correlations are presented in the results section. Statistical significance for all tests was set *a priori* at *p* ≤ 0.05.

## 3. Results

**Figure 2.**
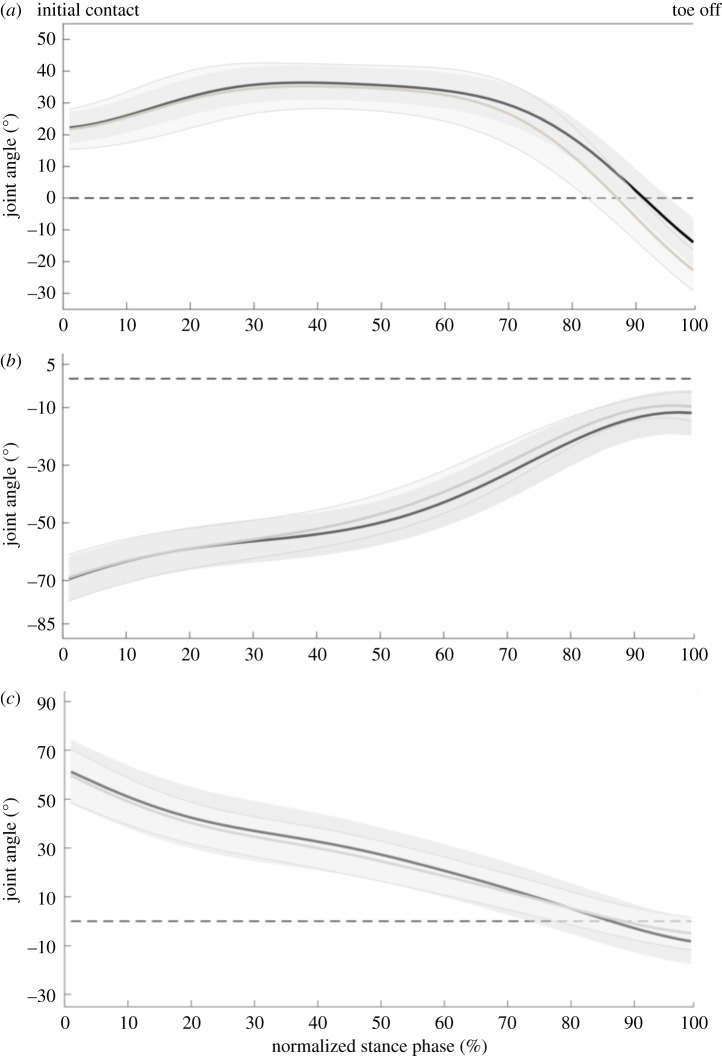
Joint angles of the ankle (*a*), knee (*b*) and hip (*c*) for the young (black line) and adult (grey line) sprinters. Shaded areas represent 1 s.d. An angle of zero degrees (dotted horizontal line) represents joint angles in the anatomical standing position.

**Figure 3.**
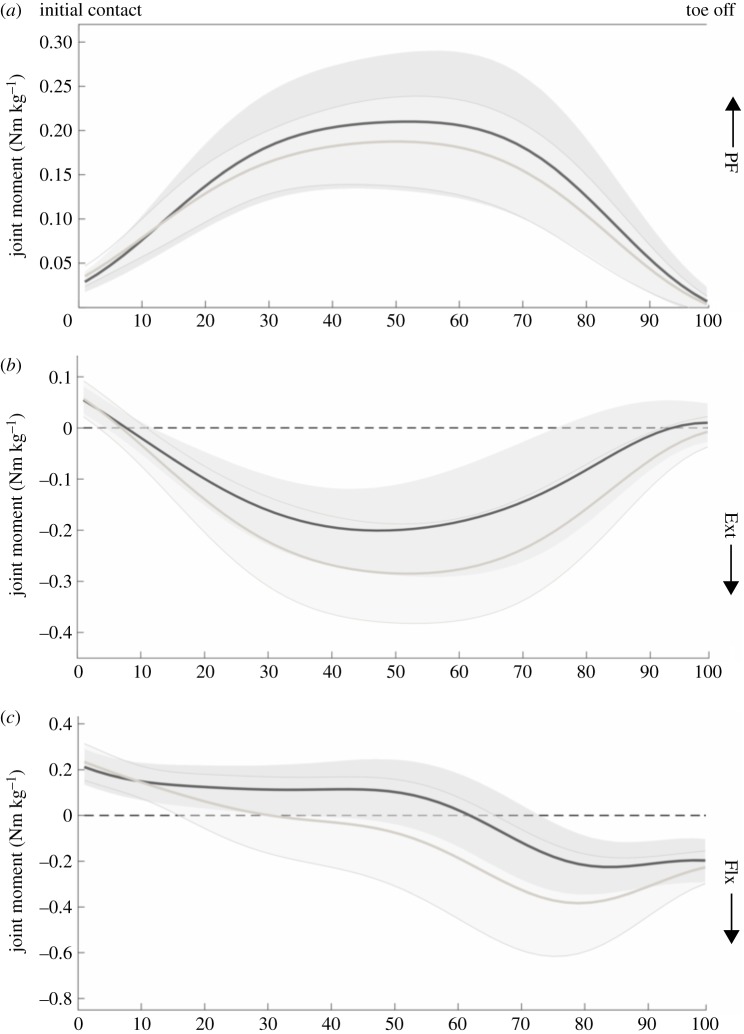
Joint moments of the ankle (*a*), knee (*b*) and hip (*c*) for the young (black line) and adult (grey line) sprinters. Shaded areas represent 1 s.d.

Only parameters that showed significant differences will be discussed. All parameter values and *p*-values related to our first aim are presented in tables 1–4. In the comparison between the adult and young sprinters, the young sprinters had a higher maximal and mean RF compared with the adult sprinters (figure 1). The maximal and mean horizontal GRF, normalized to body mass, was also higher in the young sprinters. Young sprinters also had a lower maximal angle during plantar flexion and a lower plantar flexion ROM compared with the adult group (figure 2). A lower maximal knee extensor and maximal hip flexor moment were found in the young compared with the adult sprinters (figure 3). Ankle joint stiffness on the ascending limb was higher compared with the descending limb in both groups.

**Table 1.** Performance parameters (mean ± s.d.). COM, centre of mass. Values in italics are significant *p*-values (<0.05).

parameter	young	adult	*p*-value
change in horizontal COM velocity (m s^–1^)	1.09 ± 0.25^a^	0.82 ± 0.39	*0.013*
contact time (s)	0.199 ± 0.023	0.191 ± 0.024	0.283

^a^Different from adult (*p* < 0.05).

**Table 2.** Ratio of forces (RF) in percentage of horizontal to resultant ground reaction force (GRF), non-normalized and normalized (to body mass) absolute horizontal GRF and the ankle joint stiffness on the ascending and descending limb (mean ± s.d.). *P*-values are shown for young–adult comparison and not for ascending–descending limb stiffness comparison (*p*-value for this comparison was less than 0.001). Values in italics are significant *p*-values.

parameter		young	adult	*p*-value
RF (%)	Min	−40.85 ± 20.97	−34.41 ± 27.72	0.425
	Max	78.00 ± 12.37^a^	61.31 ± 20.75	*0.004*
	Mean	37.04 ± 6.37^a^	28.49 ± 12.16	*0.009*
horizontal GRF (N)	Max	552.91 ± 147.40	488.47 ± 268.16	0.357
	Mean	333.15 ± 94.26	289.63 ± 163.32	0.314
horizontal GRF (N/kg)	Max	9.02 ± 2.00^a^	7.09 ± 3.28	*0.034*
	Mean	5.43 ± 1.28^a^	4.21 ± 2.04	*0.031*
ankle joint stiffness (Nm/deg)	Ascending	7.35 ± 3.12^b^	6.64 ± 2.01^b^	0.430
	Descending	2.85 ± 1.23	2.27 ± 0.62	0.091

^a^Different from adult (*p* < 0.05).

^b^different from descending limb (*p* < 0.05).

**Table 3.** Joint angles for the ankle, knee and hip joint in degrees (mean ± s.d.). ROM, range of motion; DF, dorsiflexion; PF, plantarflexion; Ext, extension. Values in italics are significant *p*-values.

parameter		young	adult	*p*-value
ankle (°)	Max DF	37.06 ± 5.53	36.32 ± 7.34	0.729
	ROM DF	14.87 ± 3.47	14.66 ± 2.44	0.843
	Max PF	−13.90 ± 7.68^a^	−22.73 ± 6.44	*<0*.*001*
	ROM PF	50.96 ± 9.39^a^	59.05 ± 7.40	*0*.*008*
knee (°)	Min Ext	−69.42 ± 7.80	−68.90 ± 7.97	0.843
	Max Ext	−11.19 ± 7.83	−8.81 ± 4.28	0.281
	ROM Ext	58.24 ± 6.10	60.09 ± 7.24	0.403
hip (°)	Min Ext	61.24 ± 13.27	59.56 ± 11.15	0.686
	Max Ext	−8.21 ± 9.38	−4.94 ± 6.68	0.245
	ROM	69.45 ± 9.53	64.50 ± 13.08	0.191

^a^Different from adult (*p* < 0.05).

**Table 4.** Joint moments for the ankle, knee and hip joint in degrees (mean ± s.d.). PF, plantarflexion; Ext, extension. Values in italics are significant *p*-values.

parameter		young	adult	*p*-value
ankle (Nm kg^–1^)	Max PF	0.22 ± 0.07	0.19 ± 0.05	0.262
knee (Nm kg^–1^)	Max Ext	−0.21 ± 0.09^a^	−0.29 ± 0.10	*0.015*
hip (Nm kg^–1^)	Max Ext	0.22 ± 0.07	0.24 ± 0.08	0.512
	Max Flx	−0.26 ± 0.12^a^	−0.41 ± 0.22	*0.010*

^a^Different from adult (*p* < 0.05).

**Figure 4.**
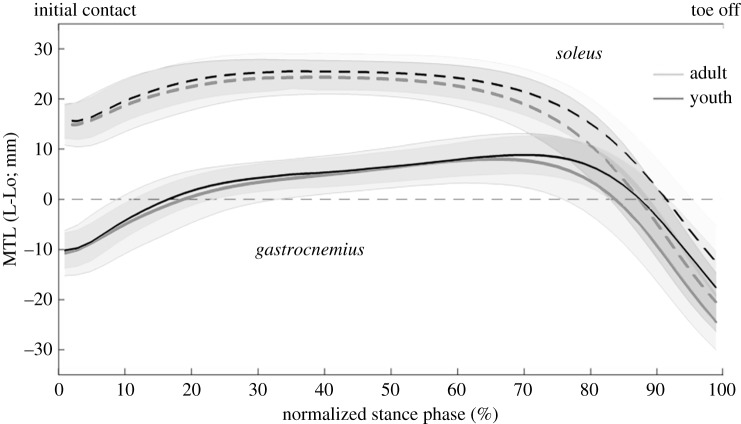
MTU lengths (MTL) of the plantar flexors for the young (black line) and adult (grey line) sprinters. For clarity, only the medial and not the lateral gastrocnemius is presented. Shaded areas represent 1 s.d. An MTL of zero mm (dotted horizontal line) represents the MTU length in the anatomical standing position (L0).

**Figure 5.**
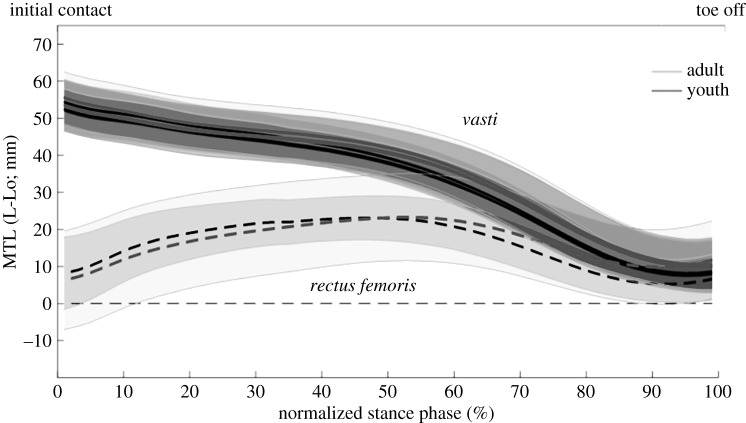
MTU lengths (MTL) of the knee extensors for the young (black line) and adult (grey line) sprinters. Shaded areas represent 1 s.d. An MTL of zero mm represents the MTU length in the anatomical standing position (L0).

The MTU length analyses showed a stretching phase followed by a shortening phase for all the plantar flexors (figure 4). In the young sprinters, the magnitude of shortening and maximal shortening velocity of the MTU were lower for all plantar flexor muscles compared with the adult sprinters (table 5). In the knee extensors, the vasti muscles presented only shortening, whereas in the bi-articular rectus femoris, a stretching phase preceded the shortening phase (figure 5). The shortening magnitude and maximal MTU shortening velocity of the rectus femoris were significantly lower in the young compared with the adult sprinters (table 6).

**Table 5.** Muscle–tendon unit (MTU) length change parameters of the plantar flexors (mean ± s.d.). Values in italics are significant *p*-values (<0.05).

MTU	parameter	young	adult	*p*-value
medial gastrocnemius	stretching (mm)	19.6 ± 3.6	19.4 ± 2.9	0.805
	stretching time (ms)	135.8 ± 33.4	116.5 ± 33.8	0.092
	shortening (mm)	27.1 ± 8.2^a^	33.2 ± 4.5	*0.011*
	shortening time (ms)	62.5 ± 25.0	73.5 ± 36.9	0.286
	maximal shortening velocity (mm ms^−1^)	0.72 ± 0.18^a^	0.85 ± 0.11	*0.017*
lateral gastrocnemius	stretching (mm)	19.5 ± 3.6	19.2 ± 2.8	0.820
	stretching time (ms)	134.9 ± 32.9	116.5 ± 33.8	0.106
	shortening (mm)	27.7 ± 8.3^a^	34.0 ± 4.5	*0.010*
	shortening time (ms)	63.2 ± 25.0	73.5 ± 36.9	0.320
	maximal shortening velocity (mm ms^−1^)	0.74 ± 0.18^a^	0.87 ± 0.11	*0.016*
soleus	stretching (mm)	10.6 ± 2.4	10.4 ± 1.4	0.784
	stretching time (ms)	73.1 ± 23.3	69.0 ± 18.5	0.563
	shortening (mm)	38.5 ± 8.3^a^	45.4 ± 5.1	*0.006*
	shortening time (ms)	122.3 ± 25.3	117.5 ± 30.1	0.603
	maximal shortening velocity (mm ms^−1^)	0.80 ± 0.17^a^	0.93 ± 0.10	*0.011*

^a^Different from adult (*p* < 0.05).

**Table 6.** Muscle–tendon unit (MTU) length change parameters of the knee extensors (mean ± s.d.). Values in italics are significant *p*-values (<0.05).

MTU	parameter	young	adult	*p*-value
rectus femoris	stretching (mm)	17.7 ± 8.4	15.8 ± 7.5	0.494
	stretching time (ms)	110.0 ± 22.2	85.3 ± 24.5	0.063
	shortening (mm)	14.9 ± 6.7^a^	19.2 ± 3.8	*0.028*
	shortening time (ms)	79.4 ± 18.0	91.5 ± 19.2	0.104
	maximal shortening velocity (mm ms^−1^)	0.27 ± 0.11^a^	0.33 ± 0.06	*0.037*
vastus lateralis	shortening (mm)	44.2 ± 4.9	45.3 ± 4.8	0.484
	shortening time (ms)	192.4 ± 24.7	176.8 ± 24.1	0.062
	maximal shortening velocity (mm ms^−1^)	0.48 ± 0.07	0.49 ± 0.06	0.450
vastus medials	shortening (mm)	43.7 ± 5.0	44.8 ± 4.9	0.509
	shortening time (ms)	192.4 ± 24.7	176.8 ± 24.1	0.062
	maximal shortening velocity (mm ms^−1^)	0.46 ± 0.07	0.48 ± 0.06	0.461
vastus intermedius	shortening (mm)	45.7 ± 5.1	46.7 ± 5.4	0.581
	shortening time (ms)	192.4 ± 24.7	176.8 ± 24.1	0.062
	maximal shortening velocity (mm ms^−1^)	0.48 ± 0.07	0.50 ± 0.07	0.506

^a^Different from adult (*p* < 0.05).

The young sprinters had a larger change in horizontal COM velocity during the first stance phase compared with the adult sprinters. A larger change in horizontal COM velocity during the first stance phase was correlated with a more negative minimal RF, a greater maximal RF and a greater mean RF (table 7). A larger change in horizontal COM velocity during the first stance phase was also correlated with a more plantar flexed ankle joint, a greater maximal knee joint extensor moment and a greater maximal hip flexor moment. Furthermore, a larger change in horizontal COM velocity during the first stance phase was correlated with a longer stretching time in the lateral and medial gastrocnemius and with a longer stretching time and more stretching of the rectus femoris MTU.

**Table 7.** Significant Pearson correlation coefficients between tested parameters and the change in horizontal COM velocity during the first stance phase; PF, plantarflexion; Ext, extension; Flx, flexion. Values in italics are significant *p*-values (<0.05).

parameter		*R*-value	*p*-value
	Min	−0.467	*0.004*
RF	Max	0.603	*<0.001*
	Mean	0.947	*<0.001*
ankle angle	Max PF	0.410	*0.012*
knee moment	Max Ext	−0.503	*0.002*
hip moment	Max Flx	−0.834	*<0.001*
lateral gastrocnemius	stretching time	0.374	*0.023*
medial gastrocnemius	stretching time	0.381	*0.020*
	stretching	0.359	*0.023*
rectus femoris	stretching time	0.440	*0.006*

## 4. Discussion

The aim of this study was to compare the biomechanics of well-trained young and adult sprinters during the first stance phase of sprint running, with a specific emphasis on MTU behaviour. We hypothesized that the young athletes lack the technical and physical abilities critical for good performance during the first stance phase, resulting in lower RF values, lower ankle joint stiffness and an increased dorsiflexion ROM in the ankle joint, ultimately leading to a lower change in horizontal COM velocity during the stance phase. We had to reject all these hypotheses as none of these parameters were different between groups, except for the maximal and mean RF which, against expectations, showed higher values in the young athletes compared with the adults. Furthermore, the young athletes had higher changes in horizontal COM velocities during the first stance phase compared to the adult athletes. Analyses of MTU length changes also showed differences between the two groups, mainly in shortening magnitude and maximal shortening velocity in the soleus and all bi-articular MTUs that were tested. The length changing patterns, mainly of the bi-articular MTUs, were ideal for energy absorption and release, likely contributing to high power generation during the first stance phase.

The higher maximal and mean RF in the young athletes compared with the adult sprinters is indicative of a greater technical ability in the young sprinters to apply larger relative portions of the resultant external force in horizontal direction. Furthermore, a significant and high correlation was found between maximal and mean RF and the change in horizontal COM velocity. Besides a higher ratio of forces, the young athletes had significantly higher mean and maximal absolute values of horizontal GRF when normalized to body mass. Various studies have shown that this body mass-normalized horizontal GRF, but not the resultant GRF, is related to sprint running performance [5,6]. Our results are somewhat in contrast to this idea, as the adult sprinters had better 100 m times than the young athletes, yet the young sprinters did have a greater change in horizontal COM velocity during the first stance phase. This confirms the results from Morin *et al*. [6], who also did not find correlations between maximal RF and 4-s sprinting distance or maximal and mean running speed over 100 m. Yet, these authors showed that the mean RF over 4-s is related to 4-s sprinting distance or maximal running speed over 100 m. As we do not have information on the absolute running speed at the beginning or the end of the first stance phase, we cannot draw any decisive conclusions on the effect of the greater RF and horizontal GRF in the young sprinters, yet, combined, these findings imply that, although a good technical ability of applying the force in horizontal direction (and thus high RF) is linked to greater changes in the COM horizontal velocity during the first stance phase, its importance in terms of final sprinting performance appears less obvious.

Joint stiffness denotes the ability of a joint to withstand a certain load. As range of dorsiflexion is likely limited and high amounts of ankle joint moment are desired for accelerating the body, high ankle joint stiffness seems desirable. However, high magnitudes of ankle joint moment and range of dorsiflexion at one hand and low magnitudes of ankle joint moment and range of dorsiflexion on the other hand could both lead to similar stiffness values. This was seen in the stiffness on the descending limb as the plantarflexion ROM was different between adult and young sprinters, yet similar stiffness of the ankle joint was found between groups on both the ascending and descending limb of the joint moment–angle curve. Furthermore, stiffness was not related to the change in COM velocity during this phase. This is somewhat in contradiction to findings from a study by Charalambous *et al*. in 2012 as they found a positive relation between ankle joint stiffness on the ascending limb and the horizontal COM velocity at the end of the first stance phase [7]. However, these authors did not look at the change in horizontal COM velocity. As such, the relation that these authors found may have been biased by the velocity of the athlete generated during the block phase, rather than during the first stance phase. Furthermore, the magnitude of ankle joint stiffness reported by these authors [7] is almost four times higher on both the ascending and the descending limb compared to the ones presented in this paper. Discrepancies between both studies could be due to the fact that the results by Charalambous *et al*. [7] are based on a single athlete. In addition, their subject was a hurdle athlete, whereas the present investigation studied 100 m sprint athletes. Owing to the technical demands of the first ground contact after each hurdle, hurdle athletes are expected to have very high ankle joint stiffness. Based on this and other studies investigating sprinting and running activities it seems that some level of ankle joint stiffness is necessary to achieve optimal performance [15–17]. Yet, none of these studies previously investigated the first stance phase in sprint running. Furthermore, Stefanyshyn *et al*. argued that joint stiffness is dependent on the task rather than on the individual [17]. Combining this with the lack of differences between our groups and the lack of correlations with the change in horizontal COM velocity in this study, we could argue that further increasing ankle stiffness during the first stance phase is not a primary factor influencing sprinting performance.

In a simulation study, Bezodis *et al*. [8] found that decreasing the dorsiflexion ROM early in the first stance phase, exponentially increased average horizontal external COM power and linked this to increased performance. Based on these results, we expected to find differences between the young and adult sprinters and to find a significant correlation between dorsiflexion ROM and the change in COM horizontal velocity. Neither of these expected results were confirmed.

Understanding the influence of various factors such as age, strength and technique on the behaviour of the MTU can greatly help our understanding of improving performance. The specific pattern of MTU length changes in the plantar flexors is theoretically advantageous in terms of generating high power outputs. The stretch–shortening cycle found in both the gastrocnemius, soleus and rectus femoris muscles allows for the storage and release of elastic energy. More specifically, the higher stiffness of the ankle joint on the ascending limb compared with the stiffness on the descending limb, in both adult and young sprinters, suggests high amounts of energy stored in the elastic tissues of the plantar flexors. However, MTU stretching magnitudes were the same between both groups in all plantar flexor muscles as well as in the rectus femoris muscle. Yet, the energy-storing potential of an MTU, mainly comes from the decoupling of the MTU and the muscle fascicles [18]. Although we did not collect any EMG data, other studies have shown that the plantar flexor muscles are activated throughout the whole stance phase, which is likely to result in a near-isometric, or slightly stretch-inducing, contraction of the fascicles [19]. Combining this with a stretching of the MTU, allows for stretching of series elastic tissues and the storage of elastic energy [20]. However, experimental data of muscle fascicle length changes during this specific sprinting task are needed to confirm the role of MTU stretching in energy storing.

The specific pattern of individual MTU length changes is likely to have important consequences for the power output during the movement. The MTU of the gastrocnemius continues to stretch even though the mono-articular soleus muscle is already shortening (figure 2). This stretching is induced by the extension of the knee joint and allows for further storage of elastic energy in the bi-articular gastrocnemius muscle, allowing for transfer of power, generated by the knee joint to the ankle. This supports previous findings by Farris *et al*. [10] who first showed this specific pattern in squat jumping. Furthermore, the time used for stretching the MTU was positively correlated to the change in COM horizontal velocity, further identifying the stretching phase as important for explosive performance. Interestingly, the adult sprinters had a higher hip flexor moment compared with the youth sprinters and the adult sprinters shift from an extensor moment to a flexor moment much earlier compared with the young sprinters (around 30% of the stance phase compared with around 60% of the stance phase, respectively). This has important consequences for the energy-storing potential of the rectus femoris MTU, as the MTU starts shortening around 60% of the stance phase. As such, this suggests that the young sprinters are unlikely to store energy in the elastic structures of the MTU, whereas the adult sprinters might as they present a flexor moment during MTU stretching.

The change in COM horizontal velocity during the stance phase was greater in the young sprinters compared with the adult sprinters. Yet, personal best times over the 100 m sprint are notably better in the adult sprinters. As such, we could argue that the young sprinters perform equally well, or even better, in the first stance phase than the adult sprinters and differences between the two groups in final performance are likely to result from later phases in the 100 m sprint. However, the differences presented in some of the parameters in this study, may have an effect on these later phases without influencing the COM horizontal velocity during the first stance phase. Future research should aim at analysing the parameters used in this study over more phases of sprint running.

We conclude that the previously highlighted, technical performance-related parameters of the first stance phase in sprinting are not likely to explain the better 100 m sprint times in adult compared to young sprinters. Combining these results with the lower knee extensor and hip flexor moments presented here, it appears that young athletes may lack the physical, strength-related, capabilities of adult sprinters, but not the technical capabilities. Furthermore, this lack of strength may have an effect on the use of elastic energy storage and release in the MTUs which could greatly influence power output.

## Data accessibility

The authors have made the dataset supporting this manuscript available from the Dryad Digital Repository and can be accessed here: https://doi.org/10.5061/dryad.p3v3n [21].
